# A community cluster of influenza A(H3N2) virus infection with reduced susceptibility to baloxavir due to a PA E199G substitution in Japan, February to March 2023

**DOI:** 10.2807/1560-7917.ES.2023.28.39.2300501

**Published:** 2023-09-28

**Authors:** Emi Takashita, Seiichiro Fujisaki, Hiroko Morita, Shiho Nagata, Hideka Miura, Yuki Matsuura, Saya Yamamoto, Shoko Chiba, Yumiko Inoue, Iori Minami, Sayaka Yoshikawa, Seiko Yamazaki, Noriko Kishida, Kazuya Nakamura, Masayuki Shirakura, Shinji Watanabe, Hideki Hasegawa

**Affiliations:** 1 Research Centre for Influenza and Respiratory Viruses, National Institute of Infectious Diseases, Tokyo, Japan; 2 Nara Prefecture Institute of Health, Nara, Japan

**Keywords:** influenza, antiviral, resistance, baloxavir

## Abstract

A community cluster of influenza A(H3N2) caused by viruses with an E199G substitution in PA was detected in Nara, Japan, between February and March 2023. The three patients with these mutant viruses had not received antiviral treatment before specimen collection but patients in the same hospital had. The sequences of the mutant viruses were closely related, suggesting clonal spread in Nara. They showed reduced susceptibility to baloxavir in vitro; however, the clinical significance of the PA E199G substitution remains unclear.

Baloxavir marboxil, a cap-dependent endonuclease inhibitor, was approved in Japan in 2018 for the treatment and prophylaxis of influenza A and B virus infections in patients 12 years and older and children younger than 12 years weighing at least 10 kg. Since the 2017/18 influenza season, we have been monitoring the susceptibility of circulating influenza viruses to baloxavir and the neuraminidase (NA) inhibitors oseltamivir, peramivir, zanamivir, and laninamivir. Approximately 10-15% of total isolates are randomly selected and analysed. During our nationwide monitoring, we detected sporadic cases of influenza A(H1N1)pdm09 and A(H3N2) viruses with reduced susceptibility to baloxavir in the 2018/19 and 2019/20 seasons [1-3]. These viruses were detected in children younger than 10 years and possessed E23K or I38T amino acid substitutions in their polymerase acidic subunit (PA). No community clusters of such mutant viruses have been identified to date.

Here, we report a community cluster of influenza A(H3N2) virus with reduced susceptibility to baloxavir due to a PA E199G substitution.

## Detection of PA E199G mutant influenza A(H3N2) viruses

In Japan, influenza activity was low throughout the COVID-19 pandemic [4]; however, during the 2022/23 season, Japan experienced its first influenza outbreak since the 2019/20 season. Baloxavir was the most used influenza antiviral during the 2018/19 season in Japan with ca 5.3 million doses [5], but the use of baloxavir has since declined due to the detection of resistant viruses and low influenza activity. The doses of baloxavir supplied to medical institutes during the 2019/20, 2020/21, 2021/22 and 2022/23 season were 600,000, 97,000, 12,000 and 590,000, respectively [16]. Influenza A(H3N2) viruses predominated, followed by A(H1N1)pdm09 and B/Victoria-lineage viruses. 

During the season 2022/23, we isolated 12 influenza A(H3N2) viruses in Nara, a town located near Osaka and Kyoto, as part of our nationwide monitoring (Table 1). In Nara, influenza A(H3N2) viruses with haemagglutinin (HA) genes from phylogenetic clades 2a.1, 2a.1b, 2a.3a and 2b co-circulated (https://nextstrain.org/flu/seasonal/h3n2/ha/2y). In Japan, 159 (48%) of the 329 influenza A(H3N2) viruses tested in this season were found to belong to clade 2b, followed by 76 (23%) that belonged to clade 2a.3a. We found that three of the 12 influenza A(H3N2) viruses possessed an E199G substitution in their PA protein, which is associated with reduced susceptibility to baloxavir [6,7]. These PA E199G mutant viruses (A/Nara/10/2023, A/Nara/12/2023 and A/Nara/14/2023) belonging to clade 2a.3a were detected in three outpatients in their 30s, 50s and 60s who presented at the same hospital between February and March 2023 with fever over 38 °C. None of them had received baloxavir before specimen collection.

**Table 1:** Influenza A(H3N2) viruses detected in Nara, Japan, 2022/23 season (n = 12)

GISAID isolate ID	Isolate name	Onset of symptoms	Specimen collection^a^	Antiviral treatment		HA clade	PA substitution
EPI_ISL_17244510	A/Nara/1/2022	15 Dec 2022	16 Dec 2022	15 Dec 2022	Oseltamivir started	2b	None
EPI_ISL_17995551	A/Nara/3/2023	28 Jan 2023	30 Jan 2023	30 Jan 2023	Baloxavir	2a.3a	None
EPI_ISL_18030665	A/Nara/8/2023	12 Feb 2023	13 Feb 2023	13 Feb 2023	Baloxavir	2a.3a	None
EPI_ISL_18030666	A/Nara/9/2023	15 Feb 2023	15 Feb 2023	15 Feb 2023	Oseltamivir started	2a.1b	None
EPI_ISL_18246484	A/Nara/10/2023	18 Feb 2023	20 Feb 2023	20 Feb 2023	Baloxavir	2a.3a	E199G
EPI_ISL_18030667	A/Nara/11/2023	18 Feb 2023	21 Feb 2023	21 Feb 2023	Oseltamivir started	2a.1b	None
EPI_ISL_17789922	A/Nara/12/2023	20 Feb 2023	21 Feb 2023	21 Feb 2023	Oseltamivir started	2a.3a	E199G
EPI_ISL_17789923	A/Nara/13/2023	28 Feb 2023	1 Mar 2023	1 Mar 2023	Oseltamivir started	2b	None
EPI_ISL_17995552	A/Nara/14/2023	2 Mar 2023	3 Mar 2023	3 Mar 2023	Oseltamivir started	2a.3a	E199G
EPI_ISL_17995564	A/Nara/19/2023	15 Mar 2023	16 Mar 2023	16 Mar 2023	Oseltamivir started	2b	None
EPI_ISL_17995581	A/Nara/20/2023	19 Mar 2023	22 Mar 2023	None		2a.1	None
EPI_ISL_17952529	A/Nara/21/2023	8 Apr 2023	9 Apr 2023	9 Apr 2023	Baloxavir	2a.1	None

GISAID: Global Initiative on Sharing All Influenza Data; HA: haemagglutinin; PA: polymerase acidic subunit.

^a ^Nasopharyngeal swabs.

Deep sequencing analysis revealed that the PA E199G substitution was present in the specimens. No amino acid substitutions associated with reduced susceptibility to NA inhibitors were detected in the PA E199G mutant viruses.

## Frequency of the PA E199G substitution in influenza A(H3N2) viruses

To assess the frequency of the PA E199G substitution in A(H3N2) viruses, we examined 81,806 PA sequences from influenza A(H3N2) viruses between 1968 and August 2023 deposited in the GISAID EpiFlu database (https://gisaid.org) (Table 2). The PA 199E position is highly conserved and only nine (0.01%) PA E199G mutant viruses were identified worldwide: one in the 2014/15, one in the 2015/16, two in the 2018/19, four in the 2021/22 and one in the 2022/23 season (excluding the ones from Nara). For those seasons, the number of PA sequences available for this analysis was 4,218, 2,809, 9,293, 20,230 and 17,304 in the 2014/15, 2015/16, 2018/19, 2021/22 and 2022/23 seasons, respectively. The frequency of the PA E199G substitution was therefore 0.02%, 0.04%, 0.02%, 0.02% and 0.006% in these seasons, respectively. Two PA E199G mutant viruses (A/Catalonia/NSVH554225590/2022 and A/Catalonia/NSVH554225591/2022) belonging to clade 2a.1 were detected on 19 April 2022 in Catalonia, Spain. At least 96 influenza A(H3N2) viruses isolated in Catalonia in April 2022 were deposited in the GISAID EpiFlu database; among those were no other PA E199G mutant viruses. These results suggest that the frequency of the PA E199G mutant A(H3N2) viruses is low.

**Table 2:** Additional influenza A(H3N2) viruses with the PA E199G substitution retrieved from 81,806 PA sequences in the GISAID EpiFlu database^a^, 1968 to August 2023 (n = 9)

GISAID isolate ID	Isolate name	Specimen collection date	Location
EPI_ISL_235649	A/New York/WC-LVD-14–091/2014	26 Dec 2014	United States
EPI_ISL_205586	A/New Jersey/53/2015	26 Dec 2015	United States
EPI_ISL_356572	A/Caerphilly/0783/2019	23 Apr 2019	United Kingdom
EPI_ISL_390205	A/Talca/63220/2019	4 Jul 2019	Chile
EPI_ISL_13431104	A/England/220880277/2022	23 Feb 2022	United Kingdom
EPI_ISL_12339077 EPI_ISL_15938127	A/Catalonia/NSVH554225590/2022	19 Apr 2022	Spain
EPI_ISL_12339076 EPI_ISL_15938128	A/Catalonia/NSVH554225591/2022	19 Apr 2022	Spain
EPI_ISL_13895980 EPI_ISL_15620975	A/Michigan/UOM10045351706/2022	4 May 2022	United States
EPI_ISL_17368119	A/Human/New York/PV82644/2022	14 Dec 2022	United States

GISAID: Global Initiative on Sharing All Influenza Data; PA: polymerase acidic subunit.

^a^ The number of PA sequences available for this analysis was 4,218, 2,809, 9,293, 20,230 and 17,304 in the 2014/15, 2015/16, 2018/19, 2021/22 and 2022/23 seasons, respectively.

For the sequences from Nara, please see Table 1.

## A community cluster of the PA E199G mutant influenza A(H3N2) viruses

Although the frequency of the PA E199G mutant A(H3N2) viruses is low, we found that three of 12 influenza A(H3N2) viruses isolated in Nara possessed the PA E199G substitution. No epidemiological link among the patients infected with the PA E199G mutant viruses could be identified, and none of them had received baloxavir before specimen collection. The whole genome sequences of these mutant viruses were identical except for an R723L substitution in polymerase basic protein 1 (PB1) of A/Nara/12/2023, suggesting clonal spread in Nara. These results indicate a community cluster of the PA E199G mutant A(H3N2) viruses in Nara.

## Antiviral susceptibility of the PA E199G mutant influenza A(H3N2) viruses

We determined the susceptibilities of the three PA E199G mutant A(H3N2) viruses to baloxavir and NA inhibitors (oseltamivir, peramivir, zanamivir and laninamivir) by using a focus reduction assay and a fluorescence-based NA inhibition assay, respectively, as previously described [8]. Our results are expressed as 50% inhibitory concentration (IC_50_) values. To interpret the antiviral susceptibility, we applied the World Health Organization criteria, which are based on fold-changes in IC_50_ values compared with the median value for viruses from the same type/subtype/lineage [5]. For NA inhibitors, normal (< 10-fold increase), reduced (10–100-fold increase) or highly reduced (> 100-fold increase) inhibition of influenza A viruses is defined [5]. For baloxavir, the provisional criteria define influenza virus inhibition as normal (< 3-fold increase) or reduced (≥ 3-fold increase). 

As shown in Table 3, the PA E199G mutant viruses showed normal inhibition with all four NA inhibitors but exhibited 3.4- to 7.3-fold higher IC_50_ values to baloxavir compared with the median IC_50_ values of wild-type A(H3N2) viruses isolated in the 2022/23 season in Japan. In a previous study, similar to our results, the PA E199G mutant A(H3N2) virus showed 4.5-fold higher IC_50_ values to baloxavir [6]. These results indicate that the PA E199G mutant A(H3N2) viruses have reduced susceptibility to baloxavir but remain susceptible to NA inhibitors.

**Table 3:** Antiviral susceptibility of influenza A(H3N2) viruses detected in Nara, Japan, 2022/23 season (n = 12)

PA substitution	Isolate name	IC_50_ (nM)				
		Baloxavir^a^	Neuraminidase inhibitors^b^			
			Oseltamivir	Peramivir	Zanamivir	Laninamivir
E199G	A/Nara/10/2023	14.90 ± 4.78	0.21 ± 0.01	0.07 ± 0.01	0.52 ± 0.03	0.70 ± 0.02
E199G	A/Nara/12/2023	31.44 ± 13.82	0.19 ± 0.01	0.06 ± 0.01	0.35 ± 0.01	0.50 ± 0.03
E199G	A/Nara/14/2023	16.57 ± 4.10	0.25 ± 0.01	0.07 ± 0.01	0.64 ± 0.06	0.59 ± 0.10
None	A/Nara/1/2022	3.55 ± 0.23	0.30 ± 0.01	0.06 ± 0.01	0.55 ± 0.01	0.56 ± 0.03
None	A/Nara/3/2023	4.56 ± 1.82	0.22 ± 0.02	0.07 ± 0.01	0.59 ± 0.02	0.67 ± 0.02
None	A/Nara/8/2023	5.41 ± 0.54	0.21 ± 0.01	0.07 ± 0.01	0.50 ± 0.02	0.62 ± 0.02
None	A/Nara/9/2023	3.97 ± 1.13	0.19 ± 0.01	0.10 ± 0.01	0.55 ± 0.02	0.79 ± 0.02
None	A/Nara/11/2023	3.59 ± 1.85	0.24 ± 0.01	0.08 ± 0.01	0.86 ± 0.02	0.79 ± 0.01
None	A/Nara/13/2023	6.21 ± 2.05	0.20 ± 0.01	0.06 ± 0.01	0.40 ± 0.01	0.54 ± 0.02
None	A/Nara/19/2023	1.60 ± 0.73	0.18 ± 0.01	0.06 ± 0.01	0.55 ± 0.03	0.68 ± 0.04
None	A/Nara/20/2023	2.52 ± 1.00	0.27 ± 0.02	0.06 ± 0.01	0.80 ± 0.05	0.77 ± 0.05
None	A/Nara/21/2023	2.78 ± 0.93	0.27 ± 0.01	0.06 ± 0.01	0.60 ± 0.03	0.62 ± 0.04
None	A(H3N2) viruses in the 2022/23 season in Japan^c^	4.33 ± 2.45	0.29 ± 0.10	0.09 ± 0.03	0.88 ± 0.33	0.93 ± 0.18

IC_50_: 50% inhibitory concentration; PA: polymerase acidic subunit.

^a^ Mean IC_50_ values of triplicate reactions were determined by use of a focus reduction assay.

^b^ Mean IC_50_ values of triplicate reactions were determined by use of a fluorescence-based neuraminidase inhibition assay.

^c^ Median IC_50_ values of influenza A(H3N2) viruses without amino acid substitutions associated with reduced susceptibility to baloxavir isolated in the 2022/23 influenza season in Japan (Baloxavir, n = 267; Neuraminidase inhibitors, n = 278).

## Discussion

The PA E199G mutant influenza A(H3N2) virus was previously detected during treatment-emergent variant monitoring in a paediatric study of baloxavir and showed reduced susceptibility to baloxavir [6]. In the study presented here, three PA E199G mutant A(H3N2) viruses (A/Nara/10/2023, A/Nara/12/2023 and A/Nara/14/2023) belonging to clade 2a.3a were detected between 20 February and 3 March 2023 in three patients without prior baloxavir treatment. The primary case of these mutant virus infections could not be identified. However, two wild-type influenza A(H3N2) viruses (A/Nara/3/2023 and A/Nara/8/2023) belonging to the same clade were detected between 30 January and 13 February 2023 in patients who received baloxavir on the day of specimen collection in the same hospital as the patients with the PA E199G mutant virus (Table 1). The whole genome sequences of A/Nara/3/2023, isolated from a teenage child, and the three PA E199G mutant viruses were almost identical except for HA I156M and NA S335G substitutions of A/Nara/3/2023. However, A/Nara/8/2023, isolated from an adult patient, possessed six substitutions compared with the PA E199G mutant viruses: two in polymerase basic protein 2 (PB2), one in PB1, two in PA and one in HA. 

An influenza outbreak occurred in the school attended by the patient infected with A/Nara/3/2023. We previously reported that influenza viruses with reduced susceptibility to baloxavir were detected 3–6 days after baloxavir administration [2]. These results suggest the possibility that the PA E199G mutant A(H3N2) virus emerged due to the selective pressure of baloxavir and started to spread earlier in the season. In addition, A/Nara/10/2023 and A/Nara/12/2023 belonged to family clusters where the transmission chain started with children, and A/Nara/14/2023 was detected in a sporadic case. As specimens from other outbreaks were unavailable, we cannot rule out that the PA E199G mutant virus circulated among children.

Of the substitutions that emerged after baloxavir treatment in the past, PA I38 substitutions were the most frequent and had the greatest impact on baloxavir susceptibility [9]. The frequency of emergence of PA I38 mutant viruses in children younger than 12 years is higher than that in patients aged 12–64 years [2,6,10]. We previously reported human-to-human transmission of PA I38T and PA E23K mutant viruses in children younger than 10 years [1-3], but no community clusters of these viruses have been identified. The higher frequency of antiviral-resistant influenza viruses in children appears to be associated with longer periods of viral shedding and higher viral titres than in adults [11]. In our current study, however, a community cluster of the PA E199G mutant virus was detected among patients in their 30s, 50s and 60s. Further studies are required to evaluate the replication and transmission fitness of the PA E199G mutant viruses.

Although patients infected with the PA I38 mutant viruses exhibited prolonged virus shedding, a rebound in virus titres after treatment and delayed symptom alleviation [10,12-15], the significance of the PA E199G substitution and the effectiveness of baloxavir in patients infected with the PA E199G mutant viruses have not been established. Our data suggest that the PA E199G mutant viruses have retained viral fitness and have the potential for human-to-human transmission. Furthermore, accumulation of multiple antiviral-resistant substitutions has a synergistic effect, resulting in a further reduction in the susceptibility to inhibitors [8]. Therefore, monitoring amino acid substitutions is essential to protect public health and support clinical management.

## Conclusion

The PA E199G mutant influenza A(H3N2) viruses showed reduced susceptibility to baloxavir in vitro; however, the clinical significance of the PA E199G substitution remains unclear. Therefore, baloxavir may be effective in patients infected with these mutant viruses.
